# Rhythmicity of Mood Symptoms in Individuals at Risk for Psychiatric Disorders

**DOI:** 10.1038/s41598-018-29348-z

**Published:** 2018-07-30

**Authors:** Luísa K. Pilz, Alicia Carissimi, Melissa A. B. Oliveira, Ana Paula Francisco, Raul C. Fabris, Madeleine S. Medeiros, Marina Scop, Benicio N. Frey, Ana Adan, Maria Paz Hidalgo

**Affiliations:** 1 Laboratório de Cronobiologia e Sono do Hospital de Clínicas de Porto Alegre (HCPA), Universidade Federal do Rio Grande do Sul (UFRGS), Porto Alegre, Rio Grande do Sul Brazil; 2 Programa de Pós-Graduação em Psiquiatria e Ciências do Comportamento – Faculdade de Medicina, UFRGS, Porto Alegre, Rio Grande do Sul Brazil; 3 Department of Psychiatry and Behavioural Neurosciences, McMaster University, Hamilton, ON, Canada, Mood Disorders Program and Women’s Health Concerns Clinic, St. Joseph’s Healthcare, Hamilton, ON Canada; 4 Hospital Materno Infantil Presidente Vargas, Porto Alegre, Rio Grande do Sul Brazil; 5 Department of Clinical Psychology and Psychobiology, School of Psychology, University of Barcelona, Barcelona, Spain; 6 Institute of Neurosciences, University of Barcelona, Barcelona, Spain

## Abstract

Despite emerging evidence that disruption in circadian rhythms may contribute to the pathophysiology of psychiatric disorders, there is a significant knowledge gap on the rhythmicity of psychological symptoms. Here, we aimed at investigating the rhythmicity of mood symptoms in individuals at risk for psychiatric disorders. 391 Brazilian and 317 Spanish participants completed the Self-Reporting Questionnaire-20 for non-psychotic mental disorders; the Mood Rhythm Instrument was used to assess rhythmicity of mood symptoms and the Munich ChronoType Questionnaire to assess sleep patterns. We found that the rhythmicity of specific mood-related symptoms and behaviors, particularly pessimism and motivation to exercise, were associated with being at risk for psychiatric disorders, even after controlling for sleep timing, sleep deficit, and season of data collection. We also found that the peak of some mood symptoms and behaviors were different between individuals at high vs. low risk for psychiatric disorders, with specific differences between countries. These results are consistent with previous research showing that circadian misalignment is associated with higher risk for mental health conditions. These findings also suggest that lifestyle changes preventing circadian misalignment might be useful to reduce the risk of psychiatric disorders, where cultural differences must be taken into account.

## Introduction

The large Global Burden of Disease study reported that mental health conditions are ranked amongst the leading causes of disability worldwide, with a prediction that major depression will be ranked the #1 cause of disability by 2025^1^. Consequently, there has been growing interest in the neurobiology of psychiatric disorders, which would help to identify individuals at risk, as well as improve treatment outcomes for those with established diagnoses^2,3^. Various neurobiological theories based on neurotransmitter systems, alterations in neuroendocrine and neuroimmune regulation, brain structure abnormalities, genetic and psychosocial factors, and circadian disruption have been proposed as etiological models of mental illnesses^4–6^.

Research on circadian rhythms and sleep regulation have revealed that alterations in social rhythms, rest-activity and sleep-wake cycle are typically observed across common psychiatric disorders, such as depression, anxiety and psychotic disorders^7–11^. In addition, several chronobiological therapeutics, such as bright light therapy, cognitive behavioral therapy for insomnia and interpersonal and social rhythm therapy, are effective in the management of mood disorders^8,9^. Despite cumulative evidence supporting the notion that circadian rhythms disruptions contribute to the pathophysiology of mood disorders, further research is warranted to better understand the underlying mechanisms and how the two are causally interconnected^6,12,13^. For instance, a significant knowledge gap is the lack of information on the rhythmicity of mood symptoms in individuals suffering from mental disorders. One of the potential reasons for this knowledge gap is that available clinical questionnaires do not take into account whether the frequency of mood symptoms follows a rhythmic pattern or the time of the day that the mood symptoms usually peak. To fill this gap, we have recently developed the Mood Rhythm Instrument (MRI), a self-reported questionnaire that evaluates the rhythmicity of mood symptoms. It includes somatic (sleep, appetite, sexual arousal), cognitive (attention, problem-solving, alertness, concentration, memory, motivation to exercise), and affective (sadness, irritability, anxiety, self-esteem, irritability, pessimism, willingness to talk with friends in person) domains. The MRI has been validated in Brazil^14^ and Spain^15^ and is now being validated in Canada. The objective of this study was to compare the rhythmicity of mood symptoms between individuals at high vs. low risk for psychiatric disorders, as measured with the Self-Reporting Questionnaire (SRQ-20). We hypothesized that individuals at risk for psychiatric disorders would report less rhythmicity of mood symptoms.

## Results

### Sample Characteristics

Demographic characteristics of the study sample are described in Table 1. In total, 391 Brazilian and 317 Spanish participants completed the SRQ-20 questionnaires. There was a statistical difference between these populations in age, sex and season when the questionnaires were conducted; therefore, these variables were included as covariates in all statistical analyses.

**Table 1:** Sample characteristics of Brazil and Spain.

	Brazil (N = 391)	Spain (N = 317)	Test contrast, p
Sex (% female)	233 (60)	214 (68)	χ² = 4.97, p < 0.05
Age, mean ± SD	21 ± 2.4	22 ± 2.5	U = 46227, p < 0.001
Season: spring/summer, n (%)	47 (12)	217 (69)	χ² = 238.43, p < 0.001
SRQ-20: positive, n (%)	151 (39)	144 (45)	χ² = 3.34, p = 0.07
MCTQ variables: median [IQR]			
MSW	03:19 [0:57]	04:07 [1:13]	U = 30469, p < 0.001
MSF	05:45 [2:12]	05:52 [1:42]	U = 53435, p = 0.14
Social jetlag	02:25 [1:45]	01:45 [1:18]	U = 74008, p < 0.001
SDw	06:30 [1:30]	07:25 [2:00]	U = 34873, p < 0.001
SDf	08:40 [1:40]	09:00 [1:55]	U = 46561, p < 0.001
Sleep deficit	02:00 [2:00]	01:30 [1:45]	U = 67107, p < 0.001
	Rhythmic (yes), n (%)		χ², p
MRI items			
Alertness	344 (88)	215 (69)	38.74, p < 0.001*
Sleepiness	374 (96)	305 (97)	0.65, p = 0.42
Problem solving	274 (70)	215 (68)	0.39, p = 0.53
Self-esteem	169 (43)	111 (35)	4.77, p < 0.05
Concentration	345 (88)	282 (89)	0.20, p = 0.66
Appetite	335 (86)	281 (89)	2.29, p = 0.13
Sexual arousal	166 (43)	165 (52)	6.43, p < 0.05
Irritability	243 (62)	201 (64)	0.17, p = 0.68
Anxiety	190 (49)	100 (32)	21.02, p < 0.001*
Sadness	162 (41)	95 (30)	9.74, p < 0.01
Motivation to exercise	274 (70)	250 (80)	8.31, p < 0.01
Memory	167 (43)	153 (48)	2.45, p = 0.12
Pessimism	135 (35)	98 (31)	1.02, p = 0.31
Talking to friends	156 (40)	181 (57)	21.98, p < 0.001*
General motivation	325 (83)	178 (57)	59.52, p < 0.001*

Chi-square (χ²) or Mann-Whitney (U) was used o compare countries as appropriate. SRQ: Self-reporting questionnaire. MCTQ: Munich ChronoType Questionnaire. MSW: midpoint of sleep on workdays; MSF: midpoint of sleep on work-free days; SDw: sleep duration on workdays; SDf: sleep duration on work-free days; SDdiff: difference between sleep duration on workdays and work-free days. MRI: Mood Rhythm Instrument.

*Significant after correction for multiple testing.

The Brazilian sample reported significantly earlier midpoint of sleep on workdays (MSW), higher social jetlag, shorter sleep duration on workdays and work-free days, and greater sleep deficit compared to the Spanish sample. The proportion of SRQ positive (high risk for psychiatric disorder) was similar between countries.

In Brazil, the proportion of individuals who reported rhythmicity for alertness, self-esteem, anxiety, sadness, and general motivation was higher than in Spain, whereas the proportion of reported rhythmicity for sexual arousal, motivation to exercise, and talking to friends was higher in the Spanish sample (Table 1).

### Rhythmicity of mood symptoms and risk for psychiatric disorders

In Brazil, among the individuals at risk for psychiatric disorders, the proportion of reported rhythmicity for the following symptoms was higher: ability to solve problems; irritability; anxiety; sadness; pessimism; and preferred time to talk to friends (p < 0.05; Table S1). The proportion of subjects reporting a peak for motivation to exercise was lower among individuals at risk for psychiatric disorders (p < 0.05). In Spain, among the individuals at risk for psychiatric disorders, the proportion of subjects who reported rhythmicity for the following symptoms was higher: anxiety; sadness; pessimism and general motivation (p < 0.05; Table S1).

Logistic regression models were run to ascertain the association between the MRI items and the likelihood of being SRQ positive or negative. In Brazil, the first logistic regression model showed sadness, pessimism and motivation to exercise were significant predictors of SRQ status (R^2^ = 0.30, p < 0.001). After controlling for age, sex, mid-sleep on free days, sleep duration difference, and season of completion the MRI, rhythmicity for sadness and pessimism on the MRI remained independent predictors for being SRQ-positive in the Brazilian sample (Table 2). In addition, motivation to exercise was an independent predictor for being SRQ-negative. Other independent predictors of SRQ status were mid sleep on work-free days, sex and season of data collection (Table 2). This model explained 40% of the variance of the SRQ classification (*p* < 0.001). In Spain, the first logistic regression model indicated general motivation, pessimism and motivation to exercise as significant predictors of SRQ status (*R*^2^ = 0.17, *p* = 0.09). Pessimism was an independent predictor for being SRQ-positive, whereas general motivation and motivation to exercise were independent predictors for being SRQ-negative in Spanish sample (Table 2). Sex was the only other predictor of SRQ status in this population (Table 2). However, the overall model was not significant (R² = 0.18, p = 0.07).

**Table 2:** Binary logistic regression of positive/negative SRQ for Brazil and Spain.

	B	S.E.	Wald	p	Exp(B) (95% CI)
**Brazil (N = 373): Nagelkerke R² = 0.40, p < 0.001**					
Sadness (rhythmic)	1.31	0.29	20.71	<0.001	3.71 (2.11–6.54)
Motivation to exercise (non-rhythmic)	0.93	0.28	10.81	<0.01	2.53 (1.45–4.39)
Pessimism (rhythmic)	1.09	0.30	13.35	<0.001	2.98 (1.66–5.34)
Mid-sleep on work-free days	0.22	0.09	5.42	<0.05	1.24 (1.04–1.49)
Sleep deficit	0.10	0.08	1.58	0.21	1.11 (0.94–1.30)
Sex (female)	1.22	0.29	18.06	<0.001	3.37 (1.93–5.91)
Age	−0.02	0.05	0.15	0.69	0.98 (0.88–1.09)
Season of data collection (winter, Mar-Sep)	−1.55	0.55	7.79	<0.01	4.70 (1.59–13.92)
Constant	−4.99	1.39	12.92	<0.001	
**Spain (N = 293): Nagelkerke R² = 0.18, p = 0.07**					
General motivation (rhythmic)	0.56	0.26	4.61	<0.05	1.75 (1.05–2.92)
Motivation to exercise (non-rhythmic)	0.63	0.31	4.12	<0.05	1.89 (1.02–3.48)
Pessimism (rhythmic)	1.13	0.28	16.45	<0.001	3.11 (1.80–5.38)
Mid-sleep on work-free days	−0.02	0.10	0.06	0.80	0.98 (0.81–1.18)
Sleep deficit	−0.02	0.09	0.08	0.78	0.97 (0.81–1.17)
Sex (female)	0.87	0.30	8.61	<0.01	2.39 (1.34–4.28)
Age	0.09	0.05	3.10	0.08	1.10 (0.99–1.22)
Season of data collection (winter, Sep-Mar)	−0.09	0.28	0.09	0.76	0.92 (0.53–1.60)
Constant	−3.51	1.43	6.05	0.01	

### Peak time of the MRI items and risk for psychiatric disorders

**Figure 1:**
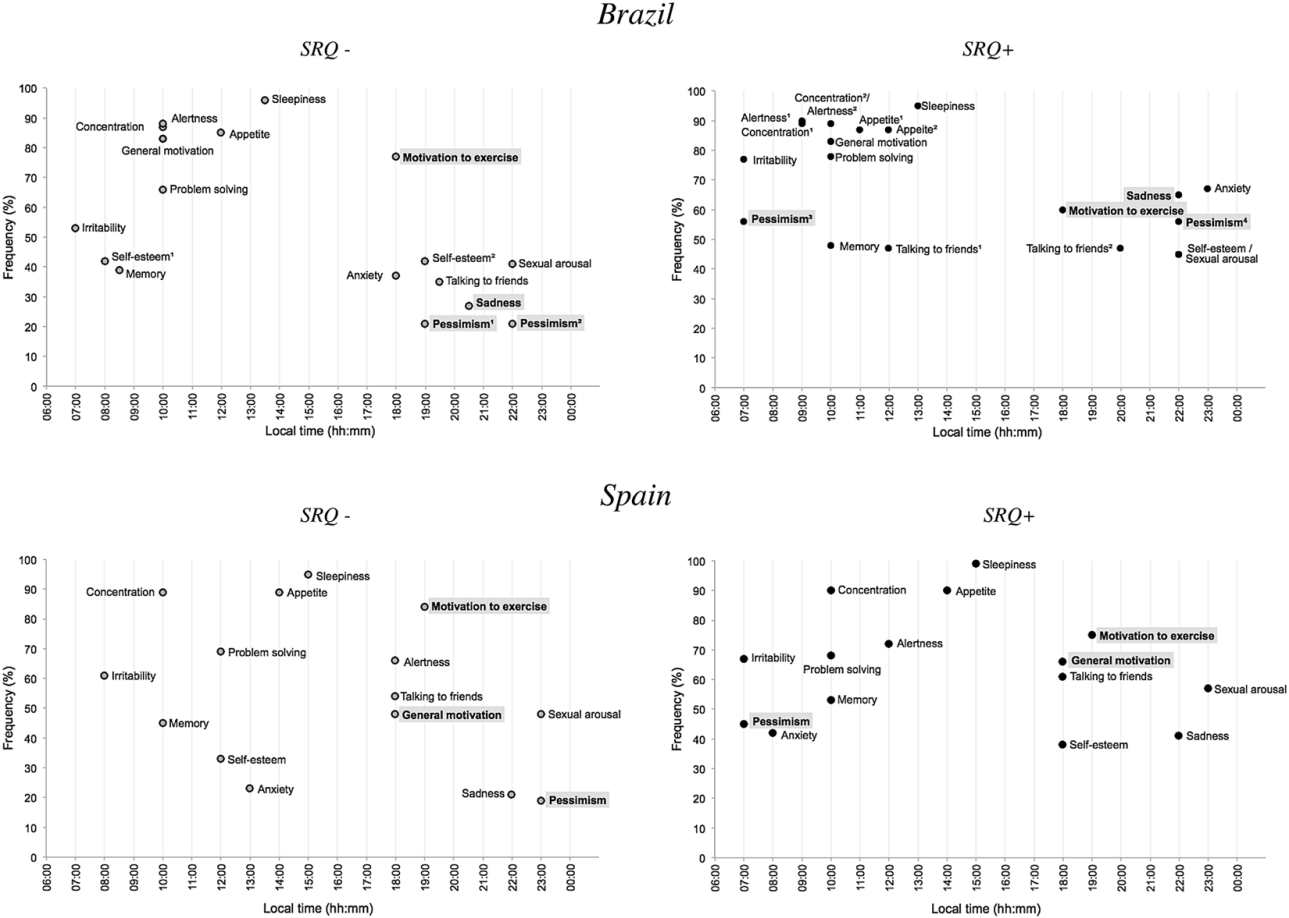
Frequency and peak time of each MRI item mode in Brazil and Spain according to SRQ status for psychiatric disorders (positive or negative). Time of day (h) is depicted on the x-axis and frequency (%) is depicted on the y-axis. Grey squares represent significant predictors of SRQ status in the binary logistic regression. ^1,2^ or ^3,4^: Bimodal variables are represented twice.

**Figure 2:**
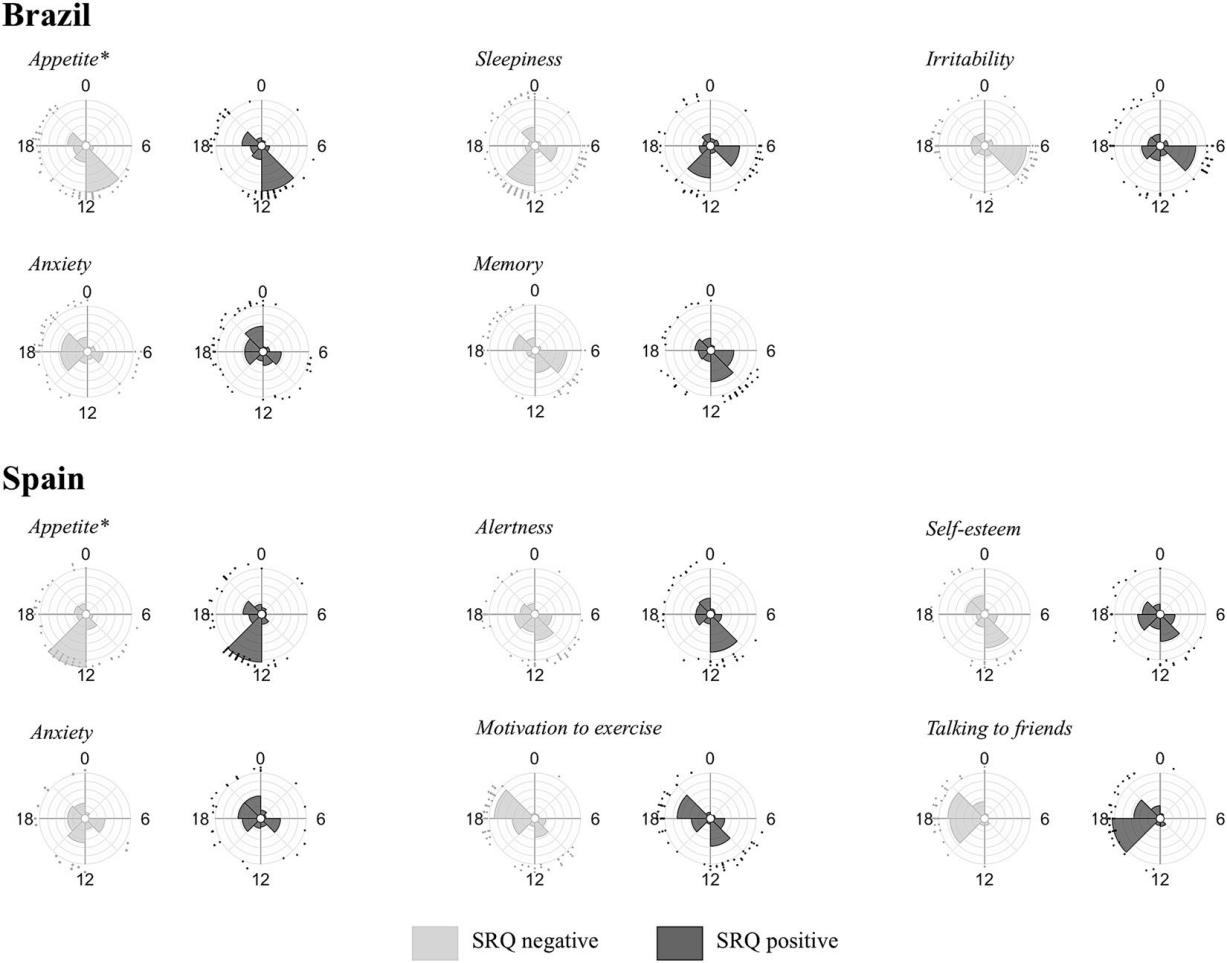
Differences between Self-reporting questionnaire (SRQ) negative and SRQ positive groups in circular distributions of the Mood Rhythm Instrument (MRI) items timing in Brazil and Spain. MRI items timing distributions are shown when they differ between SRQ negative and positive at a significance level of α = 0.30 (Mardia-Watson-Wheeler test for equal distributions). Circles represent the 24 h day. Dots along the outermost circumference represent individuals timing. Grey bars stand for the SRQ negative and black bars stand for the SRQ positive distribution. Each concentric circumference represents 10% of the sample. ^*^Appetite was significantly different between SRQ status groups at the significance level of α = 0.05 in both countries (Mardia-Watson-Wheeler test for equal distributions).

Figure 1 shows the mode of the peak of each MRI item according to country and SRQ group (SRQ positive or negative). In both countries, the distribution of peak of appetite was significantly different (Table S2). Circular distributions of the other MRI items were not different between SRQ groups; items where Mardia-Watson-Wheeler p < 0.30 are shown in Fig. 2. In Brazil, the proportion of subjects whose sleepiness peak is in the morning (5:00–12:00) was higher among the individuals at risk (*SRQ negative:* 28%/*SRQ positive:* 39%, χ² = 4.42, p < 0.05). In Spain, the phase angle difference between the peak of motivation to exercise and appetite was significantly higher in the SRQ negative group (*SRQ negative*: 2.5 [8.0]/*SRQ positive:* −1.0 [8.6], U = 3782.5, p < 0.01). The proportion of individuals who reported an earlier peak of appetite compared to the peak of motivation to exercise was higher among the SRQ positive group (SRQ *negative:* 39%/*SRQ positive:* 54%, χ² = 4.45, p < 0.05).

## Discussion

The main finding of this study was that the rhythmicity of specific mood symptoms and behaviors were strongly associated with risk for psychiatric disorders in a large sample of young adults. The perception that pessimism tends to peak at a specific time of the day was associated with high risk for psychiatric disorders, whereas the perception that the motivation to exercise tend to peak at a specific time of the day was associated with low risk for psychiatric disorders. Notably, the rhythmicity of pessimism and motivation to exercise were strongly associated with risk for psychiatric disorders in both Brazilian and Spanish samples. Considering that many psychiatric disorders are associated with pessimistic thoughts^16^, it is noteworthy that a large proportion of individuals at risk identified that they tend to feel pessimistic at a specific time of the day. Previous studies have reported higher rates of pessimism in individuals of an evening type, being sadness and pessimism among the variables that better discriminated evening from intermediate and morning types^17,18^. Thus, it could be argued that our result may be a reflection of late-types being at higher risk, since studies show higher prevalence of depression symptoms among these types^18,19^. However, the regression models indicated that the relationship between the perception of a pessimism peak and risk for psychiatric disorders was independent of sleep timing and deficit, suggesting that chronotype was not a confounder. We are unaware of any other studies that examined the relationship between the rhythmicity of mood symptoms and mental health outcomes.

The finding that individuals reporting a peak time of motivation to exercise were more likely to be screened as low risk for psychiatric disorders is consistent with previous studies showing that there is a bidirectional relationship between the circadian system and the motivation to exercise, both of which being disrupted in individuals with psychiatric disorders^20^. Exercise is an important nonphotic cue that can phase-shift circadian rhythms^21,22^ and can help re-entraining sleep-wake cycles^23^. Notably, these circadian effects are proposed mechanisms by which exercise might improve mood^24,25^. Comparing the Brazilian and Spanish samples, we found that sadness and general motivation were differentially associated with risk for psychiatric disorders. In the Brazilian sample, the frequency of rhythmicity of sadness was higher among individuals at risk for psychiatric disorders. We are unaware of previous studies that assessed the specific time of the day or the rhythmicity with which sadness may occur. It is worth noting that in our sample sadness peaked later in the day for the majority of participants. This suggests that the rhythmicity of sadness reported in this sample of young adults is different from the concept of *melancholia*, defined as “feeling worse in the morning”. Finally, feeling motivated at a specific time of the day was associated with low risk for psychiatric disorders in the Spanish sample. It has been shown that motivational behaviors have at least two components: the goal-directed or directional and the arousal or activational component^20^. Evolutionary speaking, motivational behaviors that are critical for survival such as sleeping, eating and mating cannot occur at the same time^26,27^. Thus, the circadian regulation promotes optimal timing for the various physiological needs.

We also found that the distribution of the peaks (timing) of certain mood symptoms and behaviors may differ when comparing individuals at high risk vs. low risk for psychiatric disorders. Peak of appetite, in particular, was significantly different between the two countries. Furthermore, in Brazil, the proportion of subjects whose sleepiness peak occurred in the morning was higher among individuals at risk for psychiatric disorders, which probably reflects higher rates of chronodisruption in this population. Although circular data analyses did not detect the distribution of other variables to be associated with risk for psychiatric disorders, the time relationship between items might be altered in individuals at higher risk. In Spain, the difference between the peak of appetite and motivation to exercise was significantly lower in individuals at risk, with a higher proportion of individuals at risk reporting that the peak of appetite happened earlier than the peak of motivation to exercise. This may also be a reflection of chronodisruption, since these physiological variables might have specific phase relationships^28,29^, which our results show to differ between individuals at risk or not. These findings suggest that individuals at higher risk for psychiatric disorders suffer from chronodisruption characterized by a desynchrony of biological rhythms^30^. The circadian clock is a temporal system responsible for conferring circa-24 h rhythms to physiological systems, from molecular to the behavioral level^29^. In that sense, the circadian clock orchestrates bodily functions so that they peak at optimum times during the day. Importantly, our internal rhythms need to synchronize and align with the external environment (e.g., light-dark cycle, work/school schedule, etc.), a process known as entrainment. Chronodisruption occurs when sleep/wake rhythms imposed by social schedules, such as work/school demands, result in misalignment of physiological and psychological functions. Notably, chronodisruption has been associated with a range of health issues including psychiatric disorders^5,30^.

When comparing sleep patterns across cultures, Brazilians displayed more sleep deficit, higher social jetlag, and shorter sleep duration than Spaniards. It is striking that the percentage of participants reporting less than 6 hours of sleep on workdays was 32% and 13% in Brazil and Spain, respectively. These rates are comparable to a large (n = 124,517) populational study from the US, where 15% reported sleeping less than 6 hours^31^, a known risk factor for psychiatric disorders and increased mortality^32,33^. These cultural differences may be related to how work schedules are organized since an earlier midpoint of sleep on workdays but not on free days was observed in Brazil. In Spain, most of the work activities start later than in Brazil (8/9 AM). Therefore, students and workers could delay their sleep phase without interfering with their labor activities. Considering how social routines can influence sleep/wake rhythms and may cause circadian misalignment, we hypothesize that cultural differences are essential modulators of the association between chronodisruption and risk for psychiatric disorders.

This research is a population-based cross-sectional study and, therefore, the direction of causality between MRI items and SRQ status cannot be determined. Another limitation is that we did not use a diagnostic tool for mental disorders. However, the SRQ-20 is a widely used screening questionnaire for psychiatric disorders that was tested and validated across several cultures, including Brazil and Spain^34–37^. Also, our study was mainly comprised of a homogeneous sample of young adults attending university. Thus, we cannot extrapolate our results to other populations. Despite these limitations, our relatively large and homogeneous sample allowed us to assess the association between the rhythmicity of mood symptoms and risk of psychiatric disorders, a significant knowledge gap in the interface between chronobiology and mental health research.

In conclusion, our main finding showed that the rhythmicity of specific mood-related symptoms and behaviors, particularly pessimism and motivation to exercise, were strongly associated with risk for psychiatric disorders both in Brazilian and Spanish young adults. We also found that the differences in the peak of mood symptoms and behaviors between individuals at high vs. low risk for psychiatric disorders are consistent with previous studies supporting that circadian misalignment is associated with a wide range of mental health conditions. These findings also suggest that life style changes preventing circadian misalignment might help reducing the risk of psychiatric disorders, where cultural differences must be taken into account. Future studies should try to examine how MRI may be related to objective measures of circadian rhythms, such as actimetry, cortisol or melatonin levels. Also, future studies using the MRI questionnaire in individuals with psychiatric illnesses, such as major depression and bipolar disorder, will be useful to investigate the applicability of this new clinical questionnaire to enhance the understanding of the role of chronodisruption in individuals with mood disorders.

## Methods

### Participants

In this study, 391 Brazilian and 317 Spanish participants between 18 and 29 years old were recruited. In Brazil, a sample of university students was collected. In Spain, data were collected through snowball sampling^38^. All participants gave their informed consent. The study was approved by the Ethics Committee of Hospital de Clínicas de Porto Alegre (#15-0539 GPPG/HCPA) and was conducted in accordance with the Declaration of Helsinki.

### Procedures

#### Mood Rhythm Instrument (MRI)

The MRI is a 15-item self-reported questionnaire developed by de Souza *et al*. (2016) to detect a rhythmicity of mood-related domains (affective, cognitive, somatic, physical). This scale is composed of two questions to characterize each item; the first question asks whether or not individual symptoms or behaviors had a rhythmic pattern in the last 15 days (participants report dichotomously if they had a peak for that variable - yes/no). In the second question, participants mark the exact hour in which the peak of the symptoms occurs (unimodal temporal response). The validated Portuguese^14^ and Spanish^15^ versions of the MRI were used in this study. The questionnaire demonstrated a satisfactory internal consistency (Cronbach’s alpha of 0.70 and 0.73 for Brazil and Spain, respectively^14^).

#### Munich ChronoType Questionnaire (MCTQ)

The Munich Chronotype Questionnaire (MCTQ) assesses, separately for workdays and work-free days, the time that people go to bed and the time they are ready to go to sleep, how long it takes for them to fall asleep, at what time they wake up and get up, and if they use an alarm clock^39,40^. A number of variables can be derived from the MCTQ including: the midpoint between sleep onset time and wake up time on workdays (midpoint of sleep on workdays, MSW) and on work-free days (midpoint of sleep on work-free days, MSF); social jetlag, defined as the discrepancy between the biological and social clocks calculated by the difference between MSF and MSW (social jetlag, SJL^41^); and the sleep deficit, which is calculated by the difference between sleep duration on workdays (SDw) and sleep duration on work-free days (SDf). We calculated sleep deficit as the difference between SDf and SDw.

#### Self-Reporting Questionnaire (SRQ-20)

SRQ-20 is a self-reported instrument that consists of 20 items with a yes/no answer format. It was developed by Harding and colleagues (1980) to screen for non-psychotic psychiatric disorders and was widely used in several countries and different cultures^34,35^. In this study, we used the validated Brazilian Portuguese and Spanish versions and their corresponding validated screening cut-offs to detect psychiatric disorders: scores higher than 7 were considered SRQ positive in Brazil, while scores higher than 3 were considered SRQ positive in Spain^36,37^. The Spanish and Portuguese versions of the SRQ-20 showed fair to excellent sensitivity (70%/86%) and specificity (70%/89%)^36,37^.

### Data Analysis

Chi-square tests were used to verify the relationship between dichotomous variables (i.e., SRQ positive/negative and MRI rhythmic/not rhythmic). The items that showed p < 0.20 in the chi-square were tested in a binary logistic regression. The significant predictors in the model were then tested controlling for sex, age, mid-sleep on free days and the difference of sleep duration between workdays and work-free days, and season of data collection (spring/summer or autumn/winter).

Taking into account that the variable “peak of the MRI item” is only scored when the item is considered “rhythmic”, the distribution of MRI items peaks were compared between SRQ-positive and SRQ-negative groups using the Mardia-Watson-Wheeler test. Since data are not uniform or follow a Von Mises distribution (equivalent to the normal distribution for circular data), we chose this test to examine whether the variables were distributed differently between groups^42^. Distributions of items that were different at a significance level of α = 0.30 were plotted in circular graphs.

Considering the well-established differences in circadian rhythms across cultures^43,44^, all analyses were performed separately in the Brazilian and Spanish samples. Statistical significance was set at p < 0.05. Data analyses of linear variables were performed using SPSS 24, whereas for the analyses of circular data we used NCSS 12. Circular graphs were plotted using the R package ggplot2^45^.

## Electronic supplementary material

TableS1_TableS2
