# Supplementary material for: Rhythmicity of Mood Symptoms in Individuals at Risk for Psychiatric Disorders
Source: Sci Rep. 2018 Jul 30;8:11402. doi: 10.1038/s41598-018-29348-z (PMC6065390; doi:10.1038/s41598-018-29348-z)
Supplement: Supplementary file 1 — TableS1_TableS2 [file 41598_2018_29348_MOESM1_ESM.pdf]

**Rhythmicity of Mood Symptoms in Individuals at Risk for Psychiatric Disorders**

Luísa K. Pilz, Alicia Carissimi, Melissa A. B. Oliveira, Ana Paula Francisco, Raul C. Fabris, Madeleine S. Medeiros, Marina Scop, Benicio N. Frey, Ana Adan, Maria Paz Hidalgo

Supplemental Material

TABLE S1 – Descriptive and test statistics of the proportion of rhythmic variables across SRQ + and - groups

| MRI - 15 items | Brazil | | | | | Spain | | | | |
|---|---|---|---|---|---|---|---|---|---|---|
| | Rhythmic - count (%) | | Chi-square | | | Rhythmic - count (%) | | Chi-square | | |
| | SRQ - | SRQ + | $\chi^2$ (1) | p | n | SRQ - | SRQ + | $\chi^2$ (1) | p | n |
| Alertness | 208 (87) | 136 (90) | 1.01 | 0.31 | 391 | 112 (66) | 103 (72) | 1.60 | 0.21 | 312 |
| Sleepiness | 230 (96) | 144 (95) | 0.05 | 0.82 | 391 | 164 (95) | 141 (99) | 2.69 | 0.10$^{+}$ | 315 |
| Problem solving | 157 (66) | 117 (72) | 6.71 | < 0.05$^{*}$ | 389 | 118 (69) | 97 (68) | 0.02 | 0.88 | 315 |
| Self-esteem | 101 (42) | 68 (45) | 0.29 | 0.60 | 390 | 57 (33) | 54 (38) | 0.73 | 0.39 | 315 |
| Concentration | 211 (88) | 134 (89) | 0.02 | 0.89 | 390 | 153 (89) | 129 (90) | 0.13 | 0.72 | 315 |
| Appetite | 203 (85) | 132 (87) | 0.61 | 0.44 | 391 | 152 (89) | 129 (90) | 0.14 | 0.70 | 314 |
| Sexual arousal | 99 (41) | 67 (45) | 0.60 | 0.44 | 388 | 83 (48) | 82 (57) | 2.21 | 0.14$^{+}$ | 315 |
| Irritability | 127 (53) | 116 (77) | 23.43 | < 0.001$^{*}$ | 390 | 105 (61) | 96 (67) | 1.25 | 0.26 | 315 |
| Anxiety | 90 (37) | 100 (67) | 31.43 | < 0.001$^{*}$ | 390 | 39 (23) | 61 (42) | 14.04 | < 0.001$^{*}$ | 316 |
| Sadness | 64 (27) | 98 (65) | 56.83 | < 0.001$^{*}$ | 390 | 36 (21) | 59 (41) | 15.32 | < 0.001$^{*}$ | 315 |
| Motivation to exercise | 185 (77) | 89 (60) | 14.55 | < 0.001$^{*}$ | 391 | 143 (84) | 107 (75) | 3.72 | 0.05$^{+}$ | 314 |
| Memory | 94 (39) | 73 (48) | 3.07 | 0.08$^{+}$ | 390 | 77 (45) | 76 (53) | 2.39 | 0.12$^{+}$ | 314 |
| Pessimism | 51 (21) | 84 (56) | 50.05 | < 0.001$^{*}$ | 389 | 33 (19) | 65 (45) | 25.14 | < 0.001$^{*}$ | 315 |
| Talking to friends | 85 (35) | 71 (47) | 5.20 | 0.02$^{*}$ | 391 | 93 (54) | 88 (61) | 1.63 | 0.20 | 314 |
| General motivation | 200 (83) | 125 (83) | 0.02 | 0.89 | 391 | 83 (48) | 95 (66) | 10.16 | < 0.01$^{*}$ | 314 |

$^{*}$ p < .05, $^{+}$ p < .20 (items tested in the binary logistic regression)

TABLE S2. Mardia-Watson-Wheeler test for comparing SRQ - vs. SRQ + in Brazil and Spain

| | *Brazil* | | | | | |
|---|---|---|---|---|---|---|
| | SRQ - | | SRQ + | | Mardia-Watson-Wheeler | |
| | Mode | n | Mode | n | Test stat | p |
| Alertness | 10:00 | 198 | 09:00 / 10:00 | 134 | 0.10 | 0.95 |
| Sleepiness | 13:30 | 213 | 13:00 | 136 | 2.55 | 0.28 |
| Problem solving | 10:00 | 151 | 10:00 | 115 | 0.89 | 0.64 |
| Self-esteem | 08:00 / 19:00 | 96 | 22:00 | 67 | 0.22 | 0.90 |
| Concentration | 10:00 | 201 | 09:00 / 10:00 | 130 | 1.12 | 0.57 |
| **Appetite** | 12:00 | 190 | 11:00 / 12:00 | 127 | **18.49** | **< 0.01** |
| Sexual arousal | 22:00 | 92 | 22:00 | 64 | 1.18 | 0.55 |
| Irritability | 07:00 | 127 | 07:00 | 108 | 2.70 | 0.26 |
| Anxiety | 18:00 | 87 | 23:00 | 93 | 4.88 | 0.09 |
| Sadness | 20:30 | 63 | 22:00 | 93 | 0.29 | 0.87 |
| Motivation to exercise | 18:00 | 177 | 18:00 | 88 | 1.36 | 0.51 |
| Memory | 08:30 | 87 | 10:00 | 70 | 3.85 | 0.15 |
| Pessimism | 19:00 / 22:00 | 50 | 07:00 / 22:00 | 79 | 1.08 | 0.58 |
| Talking to friends | 19:30 | 82 | 12:00 / 20:00 | 69 | 1.55 | 0.46 |
| General motivation | 10:00 | 190 | 10:00 | 122 | 0.80 | 0.67 |

| | *Spain* | | | | | |
|---|---|---|---|---|---|---|
| | SRQ - | | SRQ + | | Mardia-Watson-Wheeler | |
| | Mode | n | Mode | n | Test stat | p |
| Alertness | 18:00 | 109 | 12:00 | 103 | 2.38 | 0.30 |
| Sleepiness | 15:00 | 156 | 15:00 | 137 | 1.38 | 0.50 |
| Problem solving | 12:00 | 116 | 10:00 | 95 | 1.86 | 0.39 |
| Self-esteem | 12:00 | 56 | 18:00 | 54 | 3.92 | 0.14 |
| Concentration | 10:00 | 144 | 10:00 | 129 | 1.78 | 0.41 |
| **Appetite** | 14:00 | 141 | 14:00 | 125 | **8.33** | **< 0.05** |
| Sexual arousal | 23:00 | 73 | 23:00 | 80 | 0.78 | 0.68 |
| Irritability | 08:00 | 103 | 07:00 | 94 | 0.34 | 0.84 |
| Anxiety | 13:00 | 37 | 08:00 | 58 | 3.68 | 0.16 |
| Sadness | 22:00 | 34 | 22:00 | 58 | 0.31 | 0.86 |
| Motivation to exercise | 19:00 | 130 | 19:00 | 105 | 3.48 | 0.18 |
| Memory | 10:00 | 73 | 10:00 | 76 | 0.65 | 0.72 |
| Pessimism | 23:00 | 33 | 07:00 | 64 | 0.49 | 0.78 |
| Talking to friends | 18:00 | 84 | 18:00 | 85 | 4.54 | 0.10 |
| General motivation | 18:00 | 83 | 18:00 | 94 | 0.56 | 0.76 |
